# Binding patterns of homo-peptides on bare magnetic nanoparticles: insights into environmental dependence

**DOI:** 10.1038/s41598-017-13928-6

**Published:** 2017-10-25

**Authors:** Silvia A. Blank-Shim, Sebastian P. Schwaminger, Monika Borkowska-Panek, Priya Anand, Peyman Yamin, Paula Fraga-García, Karin Fink, Wolfgang Wenzel, Sonja Berensmeier

**Affiliations:** 1 Bioseparation Engineering Group, Department of Mechanical Engineering, Technical University of Munich, 85748 Garching b. München, Germany; 2 Institute of Nanotechnology, Karlsruhe Institute of Technology, 76344 Eggenstein-Leopoldshafen, Germany

## Abstract

Magnetic nanoparticles (MNP) are intensively investigated for applications in nanomedicine, catalysis and biotechnology, where their interaction with peptides and proteins plays an important role. However, the characterisation of the interaction of individual amino acids with MNP remains challenging. Here, we classify the affinity of 20 amino acid homo-hexamers to unmodified iron oxide nanoparticles using peptide arrays in a variety of conditions as a basis to identify and rationally design selectively binding peptides. The choice of buffer system is shown to strongly influence the availability of peptide binding sites on the MNP surface. We find that under certain buffer conditions peptides of different charges can bind the MNP and that the relative strength of the interactions can be modulated by changing the buffer. We further present a model for the competition between the buffer and the MNP’s electrostatically binding to the adsorption sites. Thereby, we demonstrate that the charge distribution on the surface can be used to correlate the binding of positively and negatively charged peptides to the MNP. This analysis enables us to engineer the binding of MNP on peptides and contribute to better understand the bio-nano interactions, a step towards the design of affinity tags for advanced biomaterials.

## Introduction

Magnetic nanoparticles (MNP) are widely used for the purification of nucleic acids and other biological molecules^1–4^. MNP are also employed in the immobilisation of enzymes^5,6^, for biomedical applications such as drug delivery and hyperthermia in cancer treatment and as contrast agents for magnetic resonance imaging^7,8^. Their superparamagnetic behaviour allows for their manipulation by an external magnetic field to easily accumulate MNP in a desired area^9^. In addition, MNP have also spiked interest in the field of catalytic reaction engineering^10^. For most applications, MNP have to be functionalised to allow for a selective binding of the target molecule. This is presently achieved by attaching metal-ion chelating molecules, e.g. nitrilotriacetic acid, to the MNP surface, which then bind His-tagged biomolecules^11,12^. Drawbacks of this method are the leakage of toxic metal ions and instability of the surface functionalisation^13,14^. Alternative surface modifications for protein adsorption on magnetic particles are glutathione, streptavidin, biotin or protein A, all of which lead to high costs running in the thousands of Euros per gram^15^. Use of bare superparamagnetic iron oxides thus offers decisive advantages for industrial applications mainly due to the easy, rapid and low-cost synthesis and the absence of degradable functional surface groups. We undertake here the first systematic study of peptide-MNP interactions of bare MNP to ultimately develop peptides that can be genetically engineered into proteins as tags and strongly bind to the nonfunctionalised MNP. The key to the design of high-affinity peptide tags lies in an in-depth understanding of surface-peptide recognition patterns^16^. However, a rational development of models guiding the design of peptides with specific binding propensity is complicated by the lack of available methods for a quantitative assessment of the affinity of individual amino acids, which are the peptide building blocks. One of the reasons that comprehensive interaction data for MNP with all the amino acids or with a larger group of peptides is currently not available may stem from the fact that MNP not only interfere with fluorescence, but also degrade fluorophores commonly used for the quantification of biomolecules^17^. In this study, we measure the interaction between bare MNP and homo-peptides using peptide arrays. Peptide arrays are commonly used in the biological context^18–22^ but have also found application in the tag development for metals and metal oxides^23–25^. This technique is particularly well suited, since iron oxide MNP stain distinctively, leaving dark spots when bound to peptides on a white cellulose membrane.

The interaction of peptides to surfaces in general is affected by many factors, such as structure^26,27^, stoichiometry^28^, pH^29^, temperature and solvent conditions^30,31^, surface protonation state^32^, possible peptide conformation^33,34^ and composition^35^. Additionally, the conformation and binding states of short peptides to metal oxide nanoparticles are influenced by pH and buffer conditions^36^. Apart from this paper, the importance of the buffer species was only recently addressed in the context of biological systems^37^, where their impact on bio-nano interactions was, however, not discussed. In comparison to homogeneous surfaces, such as carefully grown gold and platinum, or a single crystalline surface, iron oxide MNP present a particularly challenging target concerning their representation by an atomistic model, mostly due to the great heterogeneity present^38–40^. For this reason, it is difficult to utilise quantitative models based on *ab-initio* calculations that have been proven successful for other surfaces^41,42^.

In this work, we present the first systematic adsorption study of the homo-hexamers of 20 natural amino acids with magnetic nanoparticles and show clear evidence for a strong influence of the environment (buffering system) on the electrostatic characteristics of the MNP surface. It in turn leads to an observable effect on the binding of the charged peptides, while the uncharged peptides binding affinity, although present, remains untouched. For the experiments, we choose buffers with different dissociation constants, and different sign and number of charged species. We find that for certain buffer conditions, the MNP surface is not homogeneous in charge and concurrently binds peptides with different physico-chemical characteristics. In literature, various models such as the Stern-Gouy-Chapman model^43,44^, the triple-layer model (TLM)^45^, physical surface-complexion model^46^ or the charge distribution-multi site complexation (CD-MUSIC) model^47,48^ have been employed to describe similar surface systems. These models assume heterogeneous surfaces interacting with the surrounding ion layers. Some studies also included the influence of buffer ions on the adsorption of metal cations^49^. In this investigation we explore the influence of the buffer on MNP-peptide interactions and rationalise our findings by means of a model which quantitatively reproduces the change in the loading of the charged peptides on the surface for all the studied conditions. These insights enable us not only to understand interaction rules but also to develop peptides that reversibly bind MNP as a function of the buffer.

## Results

### Magnetic nanoparticles present a chimeric surface to peptides

**Figure 2:**
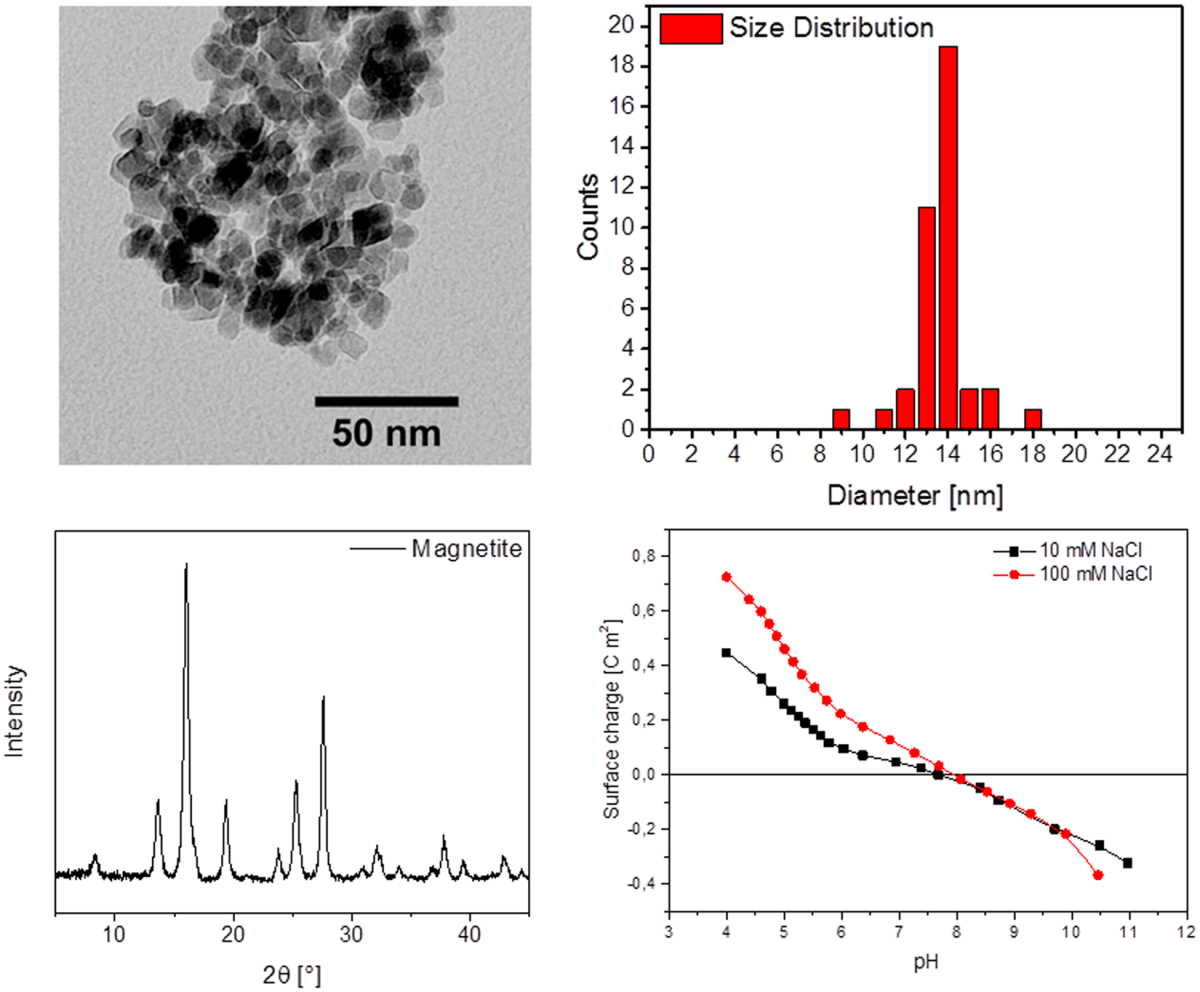
Transmission electron microscopy (TEM) image of magnetic nanoparticles used for peptide array binding experiments (top left) with corresponding particle size distribution (top right). X-ray diffractrogram of the freeze-dried nanoparticles (bottom left) and potentiometric titration (bottom right).

We first investigated the binding behaviour of the nonfunctionalised magnetite nanoparticles (MNP) to peptides at a moderately basic pH of 7.4 in tris buffered saline (TBS) solution. These particles had been extensively characterised previously with respect to their shape and size distribution by TEM and XRD (see Fig. 2 and ref.^17^) and their chemical composition by XPS, Mössbauer (See supplementary information Figure S1 and ref.^17^) and ATR-IR spectroscopy^50^. The crystalline particles present a spherical shape and a particle size of around 13.5 nm as shown in Fig. 2. The particles yield a typical spinel crystal pattern (JCPDS card no. 85-1436) with a Scherrer diameter of 8.3 nm which can also be observed in Fig. 2.

In TBS pH 7.4, zeta potential measurements indicate that the MNP surface is slightly positively charged (see Table 1). The point of zero charge was determined to be approximately 7.8 by acidometry measurements^50^. Under these conditions, one might expect that negatively charged peptides would bind to the surface with a high affinity.

**Table 1:** Zeta potentials of magnetic nanoparticles in different buffers and at different pHs used in the peptide array experiments.

Buffer	pH	Zeta potential, mV
TBS	7.4	+ 3.7 ± 0.2
	8	−5.6 ± 0.3
PBS	6	−27 ± 1.5
	7.4	−25 ± 1.3
	8	−27.8 ± 1.5
CBS	6	−34.7 ± 1.8

The values are the average of 10 measurements and an experimental error of 5.3% was determined for static zeta potential measurements.

**Figure 3:**
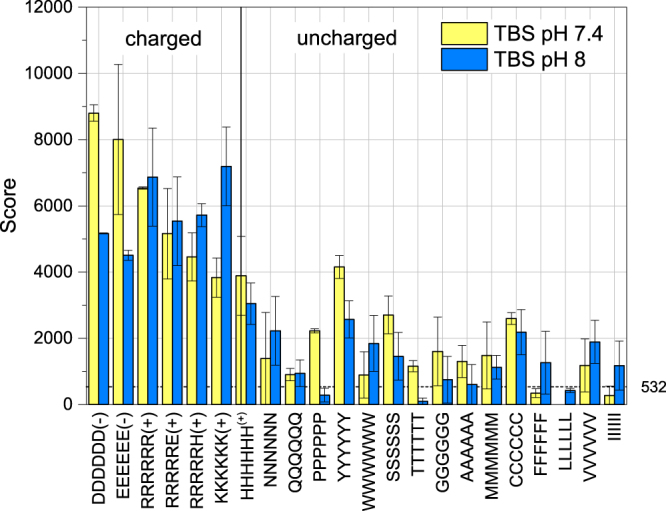
Binding scores of magnetic nanoparticles on peptides at pH 7.4 and pH 8 in TBS. The dashed line indicates the noise level in this experiment and the error bars show the standard deviation of the membrane duplicates.

In agreement with this expectation, the colorimetric data in Fig. 3, which corresponds to the absorbed mass of MNP on spots of the same size and peptide density for all experiments, show that at a pH of 7.4, the negatively charged hexa-glutamic acid (6E) and hexa-aspartic acid (6D) peptides show the highest adsorption on the MNP. Surprisingly, however, the second highest set of scores at pH 7.4 is observed for the positively charged hexa-arginine (6R), hexa-lysine (6K), followed by 5RH and 5RE, as well as hexa-histidine (6H) peptides. Histidine has a low percentage of positively charged side chains at a pH of 7.4, as indicated by its theoretical pI of 7.21 determined by the ProtParam Tool by ExPASy^51^. Histidine and carboxy groups are also known to form inner sphere complexes with metal ions^52,53^. The uncharged peptides come next although at a much lower score. Hence, the first observation is that the peptides can be categorised in their adsorption scores based on their charge. We also find that the nanoparticles do not bind strongly to the nonfunctionalised membrane seen from the low background noise level shown in the graph 3; all colorimetric scores are reported relative to this background. It is known that hydrophobic amino acids may interact with the membrane; we took care to pre-treat the membrane with methanol to minimise this effect. Next we analysed the loading of the membrane in MES buffered saline at pH 6 (see Figure S6). A similar binding pattern was obtained.

When the pH is changed to 8, the surface charge of the iron oxide nanoparticles is expected to become more negative. In accordance with this expectation, the binding scores of the negative peptides also drop from their value at the pH 7.4, while the binding scores of positive peptides rise; nevertheless, binding of both species is visible, as before. The binding of the species with the same charge polarity suggests that the nanoparticles present sites with locally varying charge polarity. Regarding the uncharged peptides, the change in the pH from 7.4 to 8 shows no distinct trend observable with regard to the experimental accuracy (Fig. 3). The lack of change in the binding scores for uncharged peptides implies that their adsorption is governed by forces that are not influenced by a change in the pH. In fact, the uncharged peptides have the same binding score regardless of the buffer solution and over the whole pH range for all the experiments performed, as seen in Figure S8 where a summary is given for the peptide interaction scores with MNP in all the different buffer conditions comparing the charged (Figure S8a) and the uncharged (Figure S8b) peptides. We clearly observe that a trend can be seen for the change in the scores of the charged peptides as a function of the buffer solution and the pH, while the uncharged peptides are unaffected with regard to these variables. As a result, within the accuracy of the measurements, the choice of buffer and the pH cannot be adequately linked to the binding affinity of the uncharged peptides, or be used to further categorise them with regard to their physical properties. Only 6P (hexa-proline) and 6Y (hexa-tyrosine) show a slight change in the score as a function of pH, but an overall rationalisation of this effect is hindered by the lack of an experimentally significant change in the rest of the (13 other) uncharged peptides.

### Buffer conditions, not only pH, control peptide affinity

**Figure 4:**
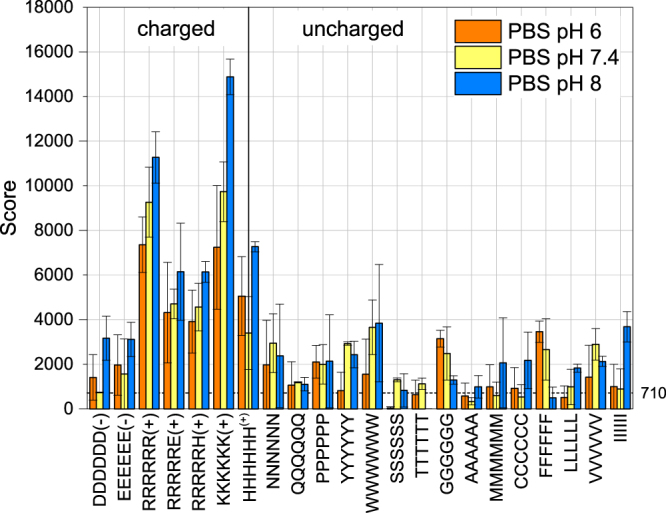
Peptide array scores of MNP at different pHs in phosphate buffered saline (PBS). The noise level is denoted by the dashed line; n = 2.

Focusing on the charged peptides we considered ways to manipulate the density of positively and negatively charged spots on the surface which are in the nanometer scale^5^. The surface composition of the MNP can be modulated during MNP synthesis or through a chemical post-synthetic modification which we wanted to avoid. An *in-situ* opportunity to modify the effective concentration of patches on the surface arises through buffer manipulation. The effective binding surface the nanoparticles present to the peptide is defined by the charge concentration in the inner Helmholtz plane, which depends on the buffer conditions (buffer species and their concentrations)^54^. Hereby, ions from the buffer compete with the (charged) groups of the peptide for adsorption. As the pK_a_ of the buffer, tris(hydroxymethyl)aminomethane (tris), is 8.07 at 25 °C^55^, a mixture of neutral and monovalent cationic tris molecules are present at the pHs of 7.4 and 8 in our experiments. Hence, to vary the number of available positive surface positions, further binding experiments were conducted in phosphate buffered saline (PBS, see Fig. 4), with phosphate present in the dissociated, anionic form at all of the aforementioned pH values (see Table 2). A closer look at the interaction score under these conditions reveals a clear trend of an increased binding of MNP to positively charged peptides with increasing pH, which is commensurate with an increase in negative charge on the MNP surface. We find that 6K scores are higher than 6R despite having a lower pK_R_ of 10.53 compared to 12.48 (Table S1)^56^. It has been argued that the charge delocalisation in the guanidinium group of arginine leads to a reduction in its hydrogen bond donating capacity to phosphate groups compared to that of lysine^57^, which may account for this observation. In contrast to the results from TBS, there is a much lower MNP adsorption on negatively charged spots, suggesting that ions in the solution compete with the peptides for nanoparticle binding. Buffer conditions can thus be varied to manipulate the local density of positive and negative binding sites. Just as in TBS, however, uncharged peptides exhibit binding which is smaller than the charged ones and is indifferent to the change of the pH.

**Table 2:** Acid dissociation constants of buffer molecules used in the peptide array binding experiments at 25 °C in aqueous solutions.

Substance	pK_a(1)_	pK_a2_	pK_a3_	Source	Charge of dominant species			Chemical structure at pH 7
					At pH 6	At pH 7.4	At pH 8	
Tris	8.07			55	+ 1	+ 1	+ 1	
Phosphate	2.16	7.21	12.33	68	−1	−2	−2	
Citrate	3.13	4.76	6.40	69	−2	−3	−3	

**Figure 5:**
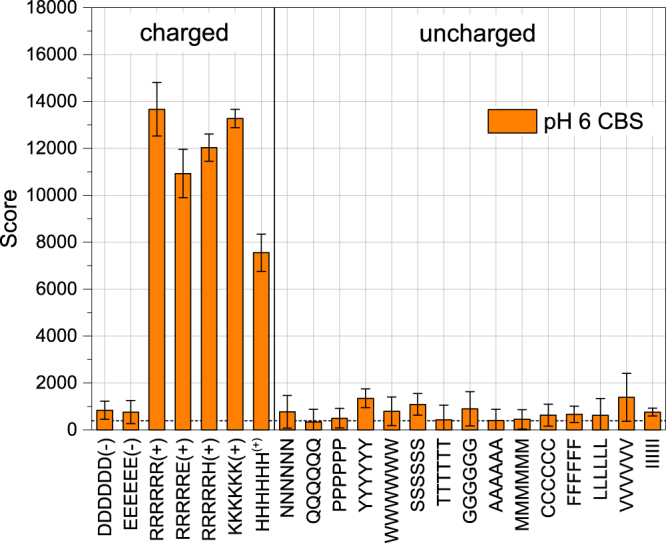
Binding scores of magnetic nanoparticles to peptides in citrate buffered saline pH 6 (CBS). The dashed line denotes the background noise level. Error bars were derived from the standard deviation of 4 measurements (two experiments were performed with membrane duplicates).

For the following tests, we chose a buffer with bivalent and trivalent anions. In citrate buffered saline (CBS) at pH 6 (Fig. 5, pk_a_ values shown in Table 2), positive peptides bind the highest quantity of magnetic nanoparticles while on negatively charged peptides no binding can be seen whatsoever. The scores for the uncharged peptides are also decreased, although remain constant from peptide to peptide. These findings are consistent with the fact that bivalent anionic buffer molecules are rather strongly adsorbed onto the iron oxide surface; this is also reflected by a change in the zeta potential from a positive value to a negative value (see Table 1 and ref.^58^). The high score of positive peptides in CBS compared to TBS and PBS at similar or higher pHs may in addition contain the effect of ionic interactions between the lysine amino groups with citrate bound to the MNP surface^59^.

### A patchwork model for the observed peptide-surface interactions

**Figure 6:**
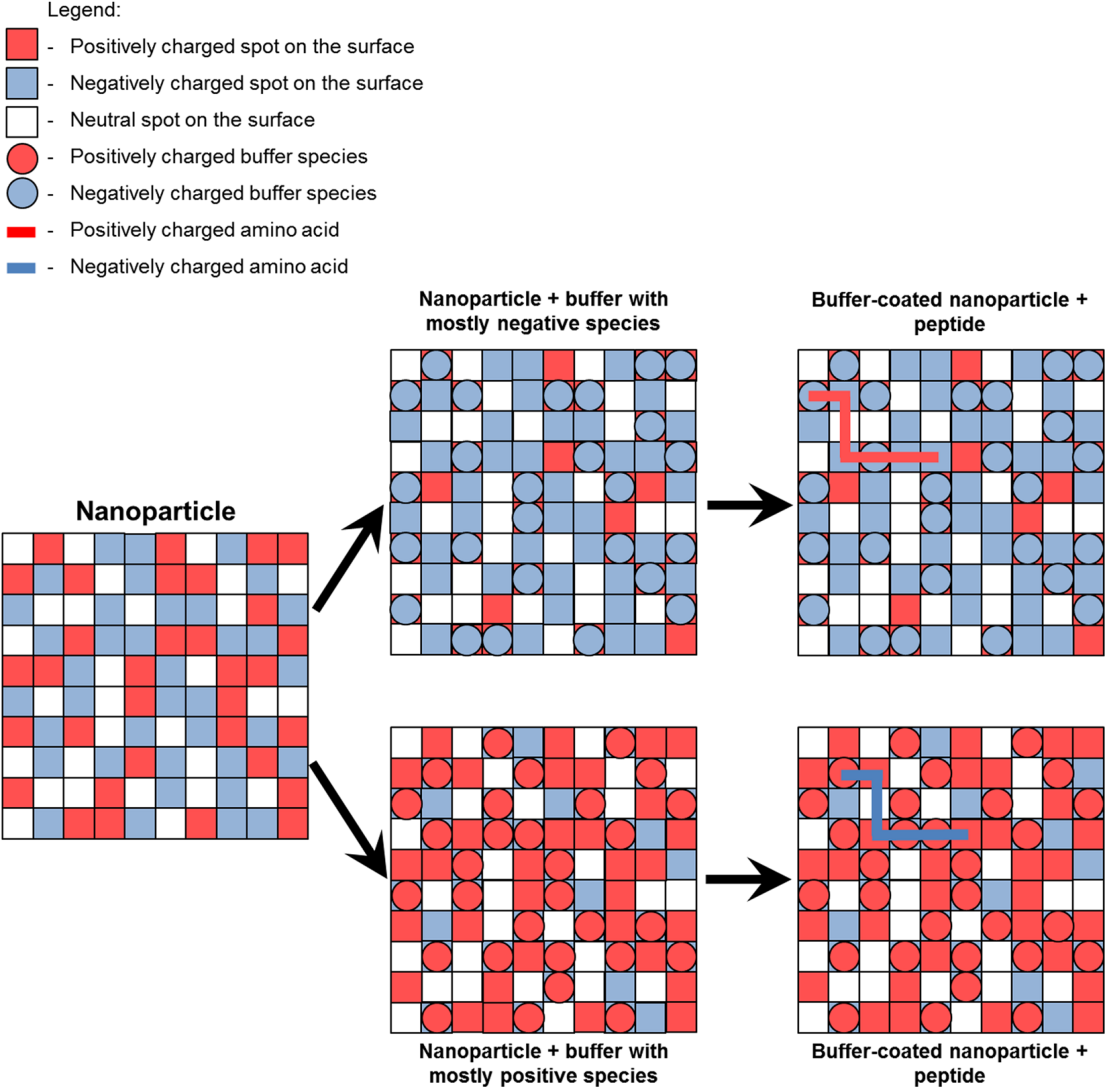
Schematic representation of a nanoparticle surface with positive, negative and neutral ‘‘spots”, denoted by red, blue and white squares, respectively. Charged buffer species are represented by red and blue circles for positive and negative charges, respectively, peptides depicted by lines. The proportion of charged spots is exaggerated for the sake of illustration.

In order to interpret the experimental results for the charged peptides, we investigate a theoretical approach to explain the diverse binding properties of peptides with different physiochemical characteristics as a function of the environmental conditions. The experimental data suggest that there is a strong interplay between the buffer conditions and the interaction strength of the charged peptides with MNP. Our model is conceptually similar to the surface complexation and adsorption models^60^, such as the multisite CD-MUSIC approach^48^. In these models, the surface is composed of a set of binding sites with a specific local chemistry that interact with ions and other adsorbates in solution. Because the loading of the surface correlates strongly with the charge of the peptides, in this investigation we perceive the bare MNP to present positive, negative and neutral binding “spots”^52,61^ (roughly the size of an amino acid^5^) that can competitively or cooperatively react with the components of the solution, as illustrated in Fig. 6. As the trends to be explained are found to be strongly related to the electrostatic nature of the species, only electrostatic interactions are considered in the model, in order to derive design rules regarding the adsorption of the charged peptides to the unfunctionalised MNP under various buffer conditions.

Due to the fact that the concentration of buffer molecules is at least an order of magnitude larger than the concentration of the magnetite nanoparticle binding sites and many orders of magnitude larger than the concentration of the peptide molecules (see Figure S4) we can use a hierarchical framework of chemical equilibria. Thereby, the buffer ions react first with the particle and the buffer composition and the pH determine the concentration of the ionic species on the surface, which is then able to bind to the peptide (Fig. 6) independent of the concentration of the peptide itself. In this way the nanoparticle is assumed to present an effective surface to the peptides defined by the buffer solution.

The spots on the surface may be considered either as a homogeneous binding site in the sense of a multisite-adsorption model^48^ or may have a size smaller than that of the peptides. Recent MD simulations support such a picture for peptide binding on titania and silica, where the peptide surface interactions are rationalised by the interactions of individual amino acids with specific surface sites, either directly, or mediated by the solvent^27,42,62–64^. Under these conditions, peptides of increasing length would need to bind to multiple spots. As positive and negative spots are present on the surface and the amino acids are constrained by peptide bonds, the peptide is presented to a large set of arrangements of positive, negative and neutral spots on the surface with a concentration that varies with pH and the buffer. However, due to the MNP surface being in a great excess compared to the peptide and hence containing a large amount of randomly distributed adsorption sites, it can be assumed that suitable patterns on the MNP surface exist for the peptides to bind. If there were not enough suitable adsorbing site patterns on the surface to accommodate the peptides, the influence of the buffer and the pH would be hindered and the observation of the significant functionalities seen from Figures 3, 4 and 5 would not be possible. Figure S9 shows that there is a size dependence of the binding score, which indicates that multiple amino acids can participate in the interaction of the peptide with the surface.

In order to model the loading on the homo-peptides, we have used the interactions of the individual amino acids with the MNP surface. We have computed the free energies of binding using all-atom peptide models within an implicit solvent/implicit surface approximation, where the interaction strength of an individual amino acid with the surface is parameterised heuristically (See SI). We note that this model does not assume any particular peptide conformation, but only that the free energy of binding of a charged peptide is proportional to the charge of the peptide and the concentration of the positive and negative spots on the MNP surface. As Figure S8 indicates for peptides of up to length eight, there is an almost linear relationship between the affinity of the peptide and its length, which is effectively the result of the lack of the influence of the conformational structure of the peptide. A similar, one-to-one relationship was observed experimentally for the tetra-to deca-homomers of aspartic and glutamic acid (Figure S9b).

**Figure 7:**
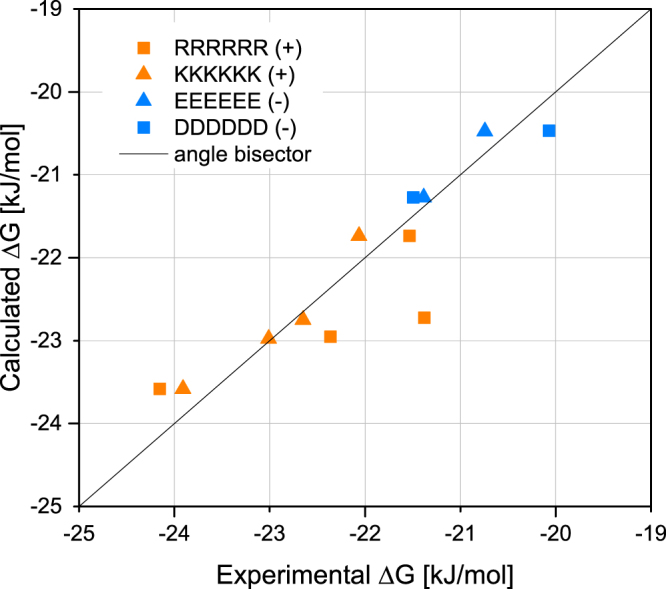
Comparison of the theoretically and the experimentally obtained binding affinities for charged homo-peptides in oppositely charged buffers and different pH. All values were recalculated to be proportional to Gibbs free energy of binding.

Based on this analysis we now test a simplified model for the binding of charged peptides to MNP in varying buffer conditions (see model section in the SI for details). The fraction of charged buffer species and the concentration of the positive and negative spots after reacting with the buffer were thus computed for all experimental situations. The fractional loading (proportional to the score of the measurements) is then correlated with the charge of the peptide. As the fractional loading is proportional to the interaction strength, the model assumes that the positive (K,R) and negative (E,D) amino acids are characterised each by one single interaction parameter. As Fig. 7 illustrates, the obtained result is consistent with the observed MNP loading of the membrane for all buffer conditions, including those with a high variety in the fractional loading. The good correlation found between the computed and the observed loadings indicates that the interaction of the peptide with the surface is largely determined by its charging interactions with the surface patches of the opposite sign. This is in line with the overall experimental finding that the buffer/pH conditions influence mostly the electrostatic interactions, thereby not influencing the uncharged peptides in a recognisable manner within the measurement accuracy. It is hence the concentration of the surface patches that is affected by the composition of the buffer.

### Reversible peptide binding

**Figure 8:**
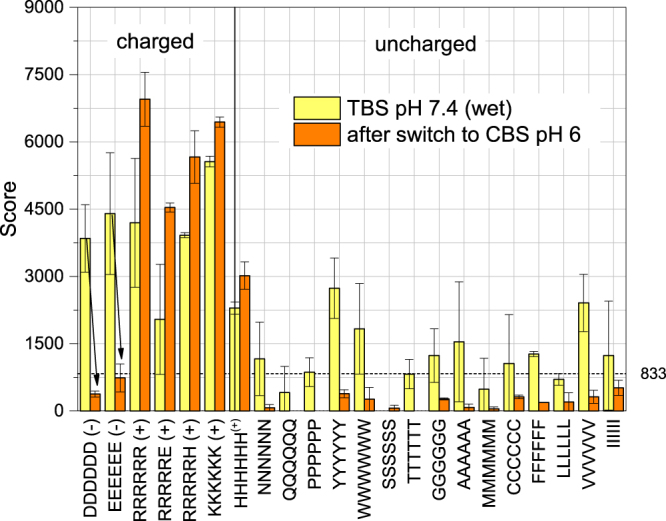
Binding scores of MNP on wet membrane after incubation in TBS pH 7.4 for 1 h and after membrane transfer to CBS pH 6 for 1 h. The noise level is represented by the dashed line. Two membrane replicates were used to obtain the data and the average and standard deviation was calculated from it.

This analysis suggests that manipulation of the buffer may be exploited for reversible peptide binding. In order to demonstrate this effect, we first prepared MNP in TBS at pH 7.4, which is favourable to bind negatively charged peptides (see Fig. 8). After binding the peptides, we washed the peptide array membrane three times with TBS and an image of the wet membrane was taken. For the other experiments the membrane had been dried before the scanning in order to increase the contrast, but this would prolong the desorption of MNP due to the need to rehydrate the particles. The wet membrane was therefore transferred to fresh CBS with a pH of 6. Under these conditions, all positively charged MNP surface sites should be covered by bivalent anionic citrate ions, which are available in excess in solution. After an incubation time of 1 h in CBS, we found that the amount of MNP on the hexa-glutamate and hexa-aspartate spots were greatly reduced showing the reversibility of the interaction. On the other hand, the binding affinity towards positive peptides increased showing a reorganisation of the nanoparticles on the array.

## Conclusions

The interaction of bare magnetic nanoparticles with all 20 natural amino acids has not previously been documented, as analytical methods struggle to quantify such affinities. By providing systematic data for homopeptides of all naturally occurring amino acids, we have shown the dependency of their interactions with non-functionalised magnetic iron oxide nanoparticles as a function of buffer solution and pH. We have demonstrated for bare MNP that chromophoric read-outs prove to be reliable for the characterisation of the interactions with short peptides. We find that both negative and positive peptides can bind the MNP while the uncharged peptides show relatively small binding propensity which is indifferent to the buffer and the pH. We therefore conclude that the electrostatic interactions play a major role in the adsorption of peptides on the MNP. Changing the buffer conditions can hence enable us to tune the interaction between the peptides and the magnetic nanoparticles. A patchwork electrostatic multisite model was used to describe the adsorption affinity (free energy) of the positive and negative peptides on oppositely charged spots on the MNP surface. The results of this analysis indicate that the binding scores of the positive and negative peptides can be fully explained by electrostatic interactions. It was further demonstrated that a reversible binding of the negative peptides to MNP can be achieved by changing the buffer conditions. These results provide a basis for the design of reversible or permanent binding tags for biomolecules that show an affinity to non-functionalised MNP. We note that this methodology is suitable to identify peptide tags for the binding of a protein to bare magnetic nanoparticles and that tag interaction patterns found in peptide arrays are in accordance with the behaviour of tagged green fluorescent protein (GFP) variants in different buffers^65^. Such tags pave the way for fast and simple immobilisation procedures without the need for unstable^13,14^ and expensive affinity ligands currently in use^15^. They therefore have the potential to be used as both efficient and effective tools for the isolation of recombinant proteins.

## Materials and Methods

All reagents used are commercially available and were used as received from the manufacturer without further purification.

### Synthesis of bare magnetic nanoparticles

The bare iron oxide nanoparticles employed for this study were synthesised by co-precipitation of Fe^2+^ and Fe^3+^ in alkaline aqueous solutions according to our previously optimised procedure^66^. 21.2 g of FeCl_3_ × 6 H_2_O and 8.3 g of FeCl_2_ × 4 H_2_O were dissolved in 200 mL of deionised, degassed water resulting in a Fe(III): Fe(II) ratio of 1.9: 1. This iron chloride solution was added to 1 L of 1 M NaOH prepared with deionised, degassed water stirring at 250 rpm in a reaction vessel. The reaction mixture was kept under a nitrogen atmosphere at 25 °C and stirred for an additional 30 minutes before the resulting nanoparticles were washed with deionised water until the conductivity of the MNP solution was below 200 µS cm^−1^. In order to separate the particles, the mixture was placed on a NdFeB permanent magnet. Suspensions were lyophilised with an ALPHA 1-2LD plus from Martin Christ Gefriertrocknungsanlagen GmbH, Germany in order to obtain solid particles. FeCl_3_ × 6 H_2_O and sodium hydroxide were purchased from AppliChem GmbH, Germany in the highest purity available. FeCl_2_ × 4 H_2_O extra pure was obtained from Merck KGaA, Germany.

### Transmission electron microscopy (TEM)

TEM images were recorded using a JEM 100-CX (JEOL GmbH, Germany). For the TEM measurements the colloidal samples were diluted in degassed and deionised water, ultrasonicated to disperse any agglomerates and precipitated on carbon coated copper grids (Quantifoil Micro Tools GmbH, Germany). The pictures were manually processed in ImageJ. 30 particles were measured in random order.

### X-ray diffraction (XRD)

Crystal structure and phase purity of the lyophilised samples were examined with powder X-ray diffraction (XRD). The measurements were performed with a Stadi-P diffractometer (STOE & Cie GmbH, Germany), equipped with a molybdenum source (λ = 0.7093 Å) and a Mythen 1 K detector (DECTRIS Ltd., Switzerland) in transmission geometry. Data was collected in the range from 2° to 50° (2θ). The software package STOE WinXPOW (STOE & Cie GmbH, Germany) was used for indexing and refinement purposes.

### Potentiometric titrations

Potentiometric titrations were accomplished in an OptiMax™ reactor (Mettler-Toledo GmbH, Germany) from pH 4 to 10. The degassed particle suspensions were adjusted to a concentration of 2 g L^**−**1^ and equilibrated at a pH of 4 overnight. The whole titration was conducted under nitrogen atmosphere at 298.5 K with HCl and NaOH as titrands and two different NaCl concentrations of 10 and 100 mmol L^**−**1^.

### Magnetic nanoparticle binding assay

**Figure 1:**
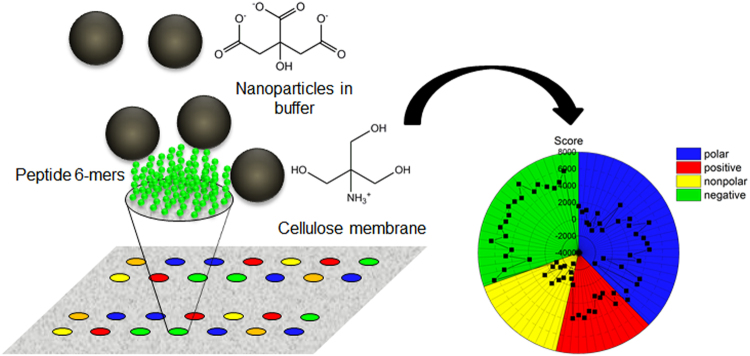
Schematic experimental setup of peptide array binding experiments and their evaluation. Peptides were immobilised on a cellulose membrane in spots. Each spot contained 5 to 10 nm of a certain peptide. The nanoparticles were suspended in buffer and added to the membrane. A higher load with particles resulted in a darker colour of the spot which was digitalised to yield a score as an indicator for their adsorption.

In order to determine the binding of peptides to bare magnetic nanoparticles (MNP) CelluSpot peptide arrays from Intavis with 5 to 10 nmol of peptides per spot were used. The peptides were bound to the cellulose membrane via the C-terminus and the free N-terminus is acetylated. The spot diameter was 2 mm. A schematic picture of the setup is shown in Fig. 1. The membrane on which the peptides had been synthesised by the manufacturer was conditioned with 1 mL of methanol in order to rehydrate hydrophobic peptides^67^. Methanol is toxic and should be handled with care. The buffers used were 50 mM of citrate (CBS), phosphate (PBS), tris(hydroxymethyl) aminomethane (TBS) and 2-(N-morpholino)ethanesulfonic acid (MES-BS) in double distilled water and were supplemented with 137 mM NaCl and 2.7 mM KCl. Finally, Tween 20 was added to a concentration of 0.25% (v/v) to the buffers to reduce nonspecific binding. The orbital shaker used for incubation was MulitBio3D from Biosan.

The array membrane was washed three times for 10 min each with 50 mL of buffer rotating of the orbital shaker at 30 rpm. After washing, the membrane was incubated for 60 minutes in a 0.4 g/L MNP suspension^25^ at the same rocking speed. The MNP suspension was freshly prepared before the experiment by adding buffer to lyophilised nanoparticles and sonicating for 15 minutes. Unbound particles were removed from the membrane by washing with buffer three times for 10 minutes. In order to test for reversible binding, the membrane was consecutively incubated in the buffer of interest for 1 h. The cellulose membrane was dried overnight at 4°C; then an image was taken using a GelDoku station. An example picture is shown in Figure S2. To quantify the staining of the spots, the microarray profile plugin for ImageJ was used. The output of this plugin is a mean value of the spot darkness, which is correlated to the amount of bound magnetic nanoparticles. An average value of the background was determined from 32 spots on the membrane without any peptides. The difference between this background value and the darkness of the peptide spots is dependent on the amount of magnetic nanoparticles that was adsorbed by the peptides and is therefore an indicator of the binding selectivity of the peptides towards the MNP. In order to determine the background noise, the standard deviation was calculated for the darkness values of these 32 spots without any peptides. For each experiment, this noise level is indicated as a dashed horizontal line in the respective figures. The membrane was regenerated in 100 mM oxalic acid for 40 minutes rotating on the orbital shaker after each use and washed for 10 minutes with double distilled water three times. Dried membranes were stored at −20°C in a sealed plastic bag.

### Zeta potential measurements

A buffered suspension of 0.4 g/L magnetic nanoparticles as used in the peptide array binding assays described above was sonicated for 15 minutes. The zeta potential was determined using the Smoluchowski equation in a Beckman Coulter Delsa Nano C at 25 °C. Each measurement was taken three times with 10 accumulations and a pinhole of 50 µm.

## Electronic supplementary material

Binding patterns of homo-peptides on bare magnetic nanoparticles: insights into environmental dependence
