# Supplementary material for: Binding patterns of homo-peptides on bare magnetic nanoparticles: insights into environmental dependence
Source: Sci Rep. 2017 Oct 25;7:14047. doi: 10.1038/s41598-017-13928-6 (PMC5656586; doi:10.1038/s41598-017-13928-6)
Supplement: Supplementary file 1 — Binding patterns of homo-peptides on bare magnetic nanoparticles: insights into environmental dependence [file 41598_2017_13928_MOESM1_ESM.pdf]

SUPPLEMENTARY INFORMATION

Binding patterns of homo-peptides on bare magnetic nanoparticles: insights into environmental dependence

Silvia A. Blank-Shim[1], Sebastian P. Schwaminger[1], Monika Borkowska-Panek[2], Priya Anand[2], Peyman Yamin[2], Paula Fraga-García[1], Karin Fink[2], Wolfgang Wenzel[2*], Sonja Berensmeier[1*]

[1]Bioseparation Engineering Group, Department of Mechanical Engineering, Technical University of Munich, 85748 Garching b. München, Germany

[2]Institute of Nanotechnology, Karlsruhe Institute of Technology, 76344 Eggenstein-Leopoldshafen, Germany

*corresponding authors: s.berensmeier@tum.de and wolfgang.wenzel@kit.edu

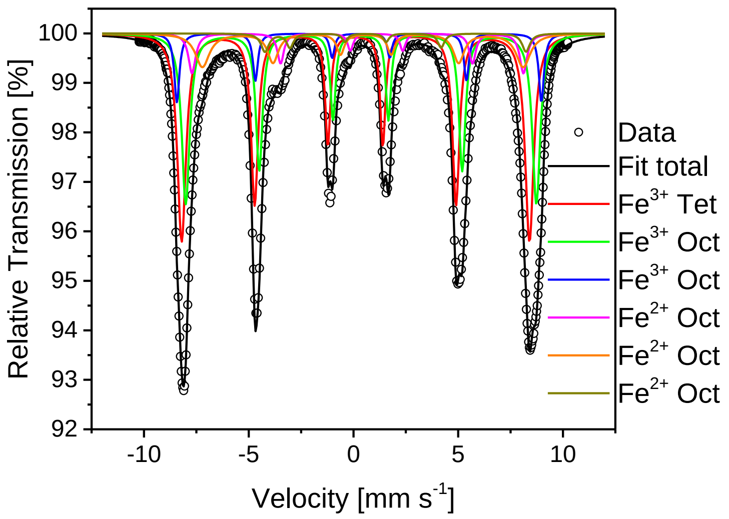

Figure S1 Mössbauer spectrum of MNPs at 4.2 K representing nanoscale magnetite.

Table S1. Chemical structures, one letter code and acid dissociation constants of side chains of selected L-amino acids[1].

Amino acid	Symbol	pK_R	Charge of dominant species at pH 6 to 8	Chemical structure at pH 7
Lysine	K	10.53	+1	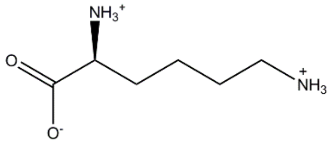
Arginine	R	12.48	+1	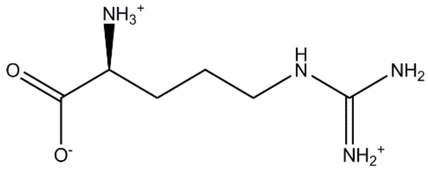
Histidine	H	6.00	0	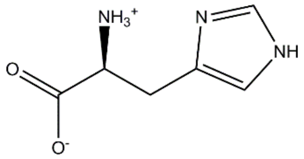
Aspartate	D	3.65	-1	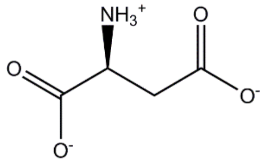
Glutamate	E	4.25	-1	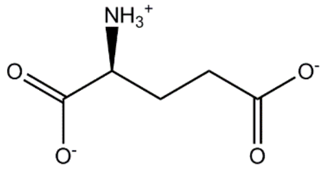

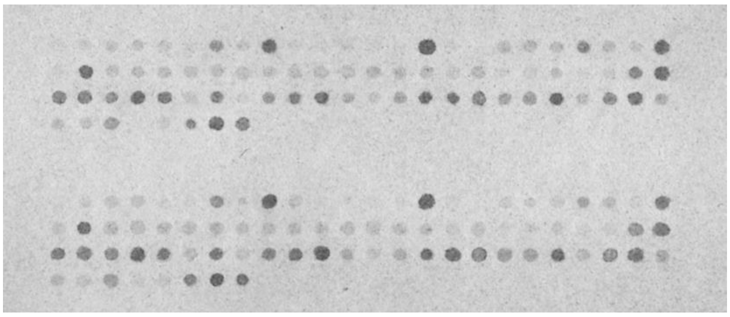

Figure S2 Picture of peptide array membrane with MNP bound to certain peptide spots.

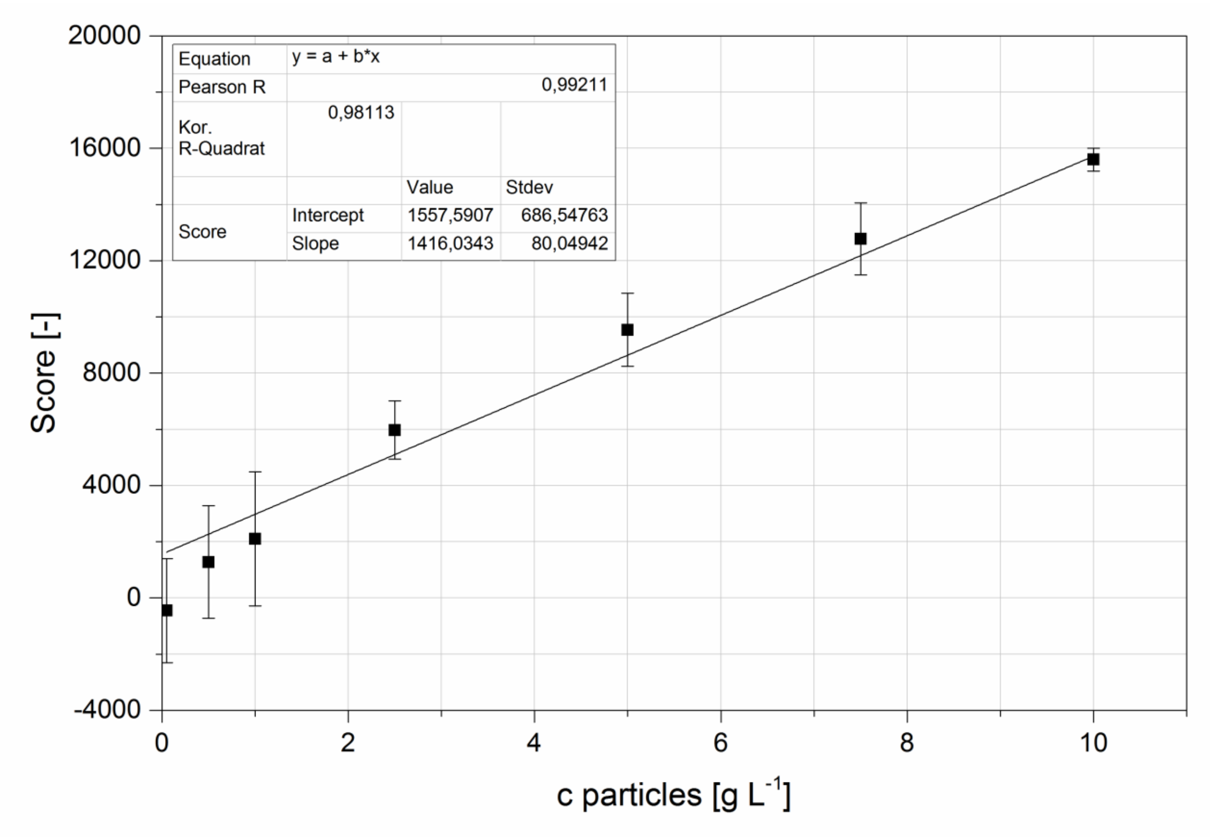

Figure S3 Calibration curve of magnetic nanoparticles on empty cellulose membrane without peptides. Bare magnetic nanoparticle suspensions with known MNP concentration was dropped onto the membrane and the darkness score determined by ImageJ similar to the evaluation of peptide array experiments.

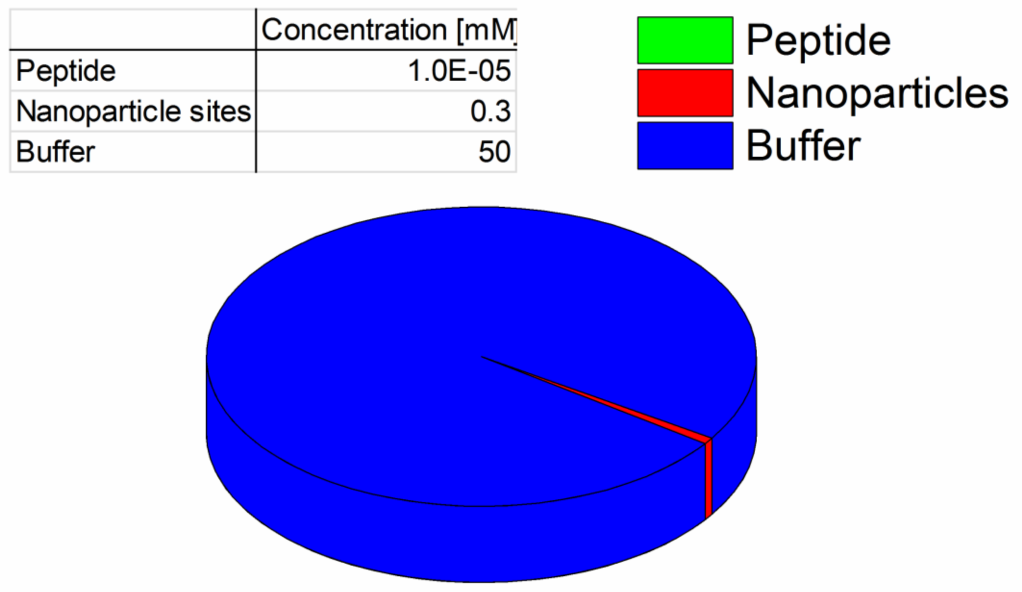

Figure S4 Concentrations of components during peptide array experiments.

Calculation of nanoparticle site concentration:

$$\sigma_o = F(\Gamma_{H^+} - \Gamma_{OH^-}) = F\frac{c_{NaOH}(v_b - v_d)}{s \cdot \gamma \cdot V}$$

$$\sigma_o = surface\ charge\ \left(\frac{C}{m^2}\right) = 0.73\ \frac{C}{m^2}$$

$$\Gamma = surface\ concentration\left(\frac{mol}{m^2}\right)$$

$$F = Faraday\ constant\ \left(\frac{C}{mol}\right) = N_A \cdot e = 96485.3\ \frac{C}{mol}$$

$$N_A = Avogadro\ constant$$

$$e = elemental\ charge$$

$$\sigma_o = surface\ charge\left(\frac{C}{m^2}\right)$$

$$V = volume\ liquid\ (l)$$

$$s = specific\ surface\ area\ \left(\frac{m^2}{g}\right) = 100\ \frac{m^2}{g}$$

$$\gamma = concentration\ particle\left(\frac{g}{l}\right)$$

$$v_b = titrated\ blank\ volume\ (l)$$

$$v_d = titrated\ base\ volume\ (l)$$

$$c_{NaOH} = concentration\ base\left(\frac{mol}{l}\right)$$

$$\sigma_{max} = F \cdot \Gamma_{H^+ max}$$

$$\Gamma_{H^+ max} = maximal\ surface\ concentration\ of\ H^+ \triangleq surface\ site\ density = \frac{\sigma_o}{F} = 7{,}57 \cdot 10^{-6}\frac{mol}{m^2} = 4{,}5\frac{sites}{nm^2}$$

$$c_{Nanoparticle\ sites} = \frac{\Gamma_{H^+ max} \cdot s \cdot \gamma}{N_A} = 0.3\ mM$$

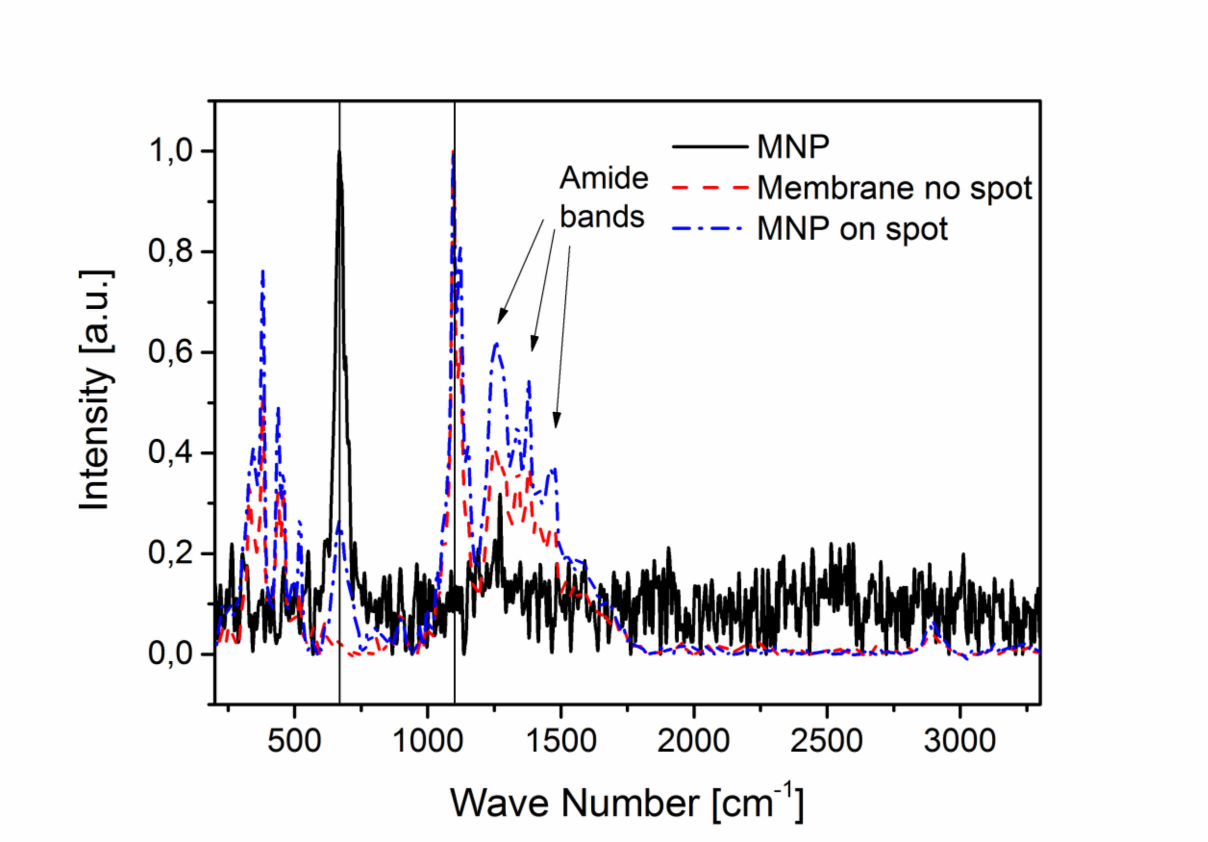

Figure S5 Normalized Raman spectra of MNP (magnetite), the bare peptide array membrane and a spot with MNPs bound.

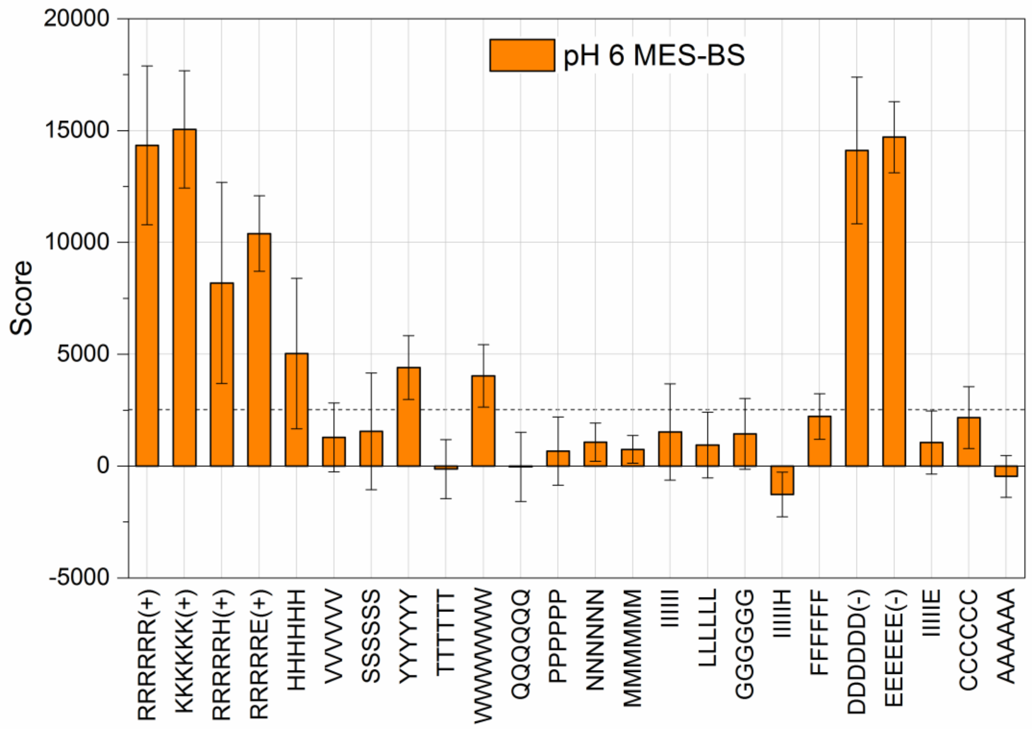

Figure S6 Interaction scores of peptides with MNP in 2-(*N*-morpholino)ethanesulfonic acid buffered saline (MES-BS) pH 6. The MNP zeta potential was -7.7 mV.

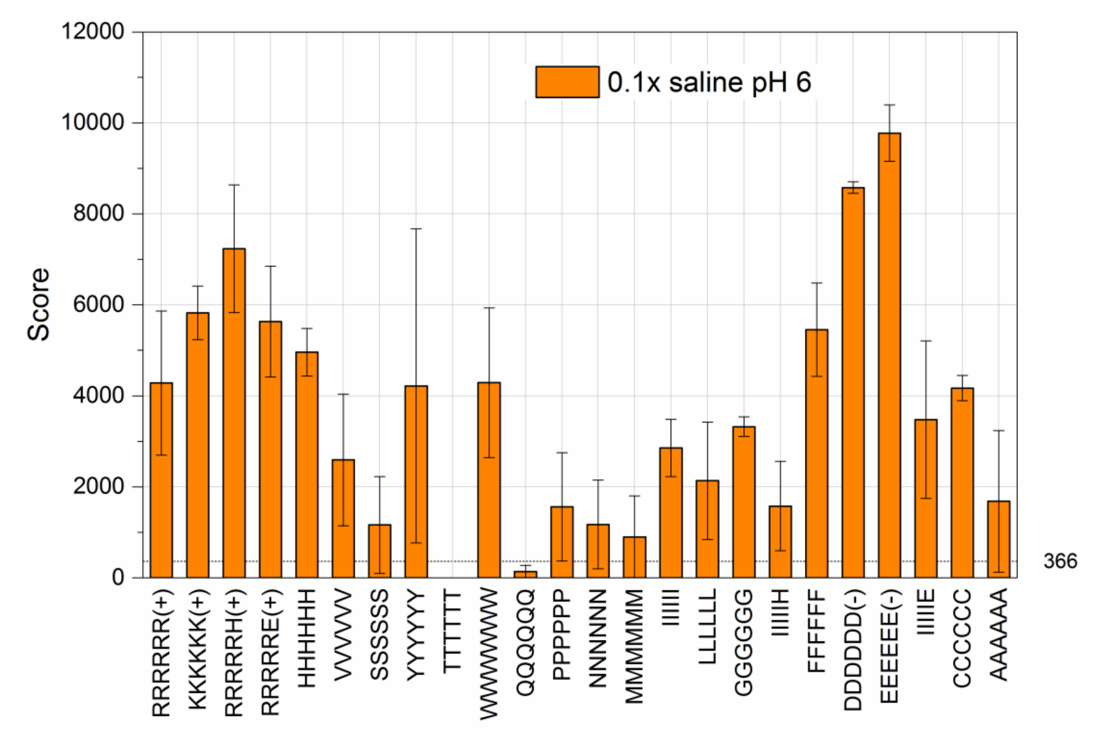

Figure S7 Binding affinities of MNP in unbuffered saline (13.7 mM NaCl + 0.27 mM KCl) pH 6 to peptides on array membrane.

a)

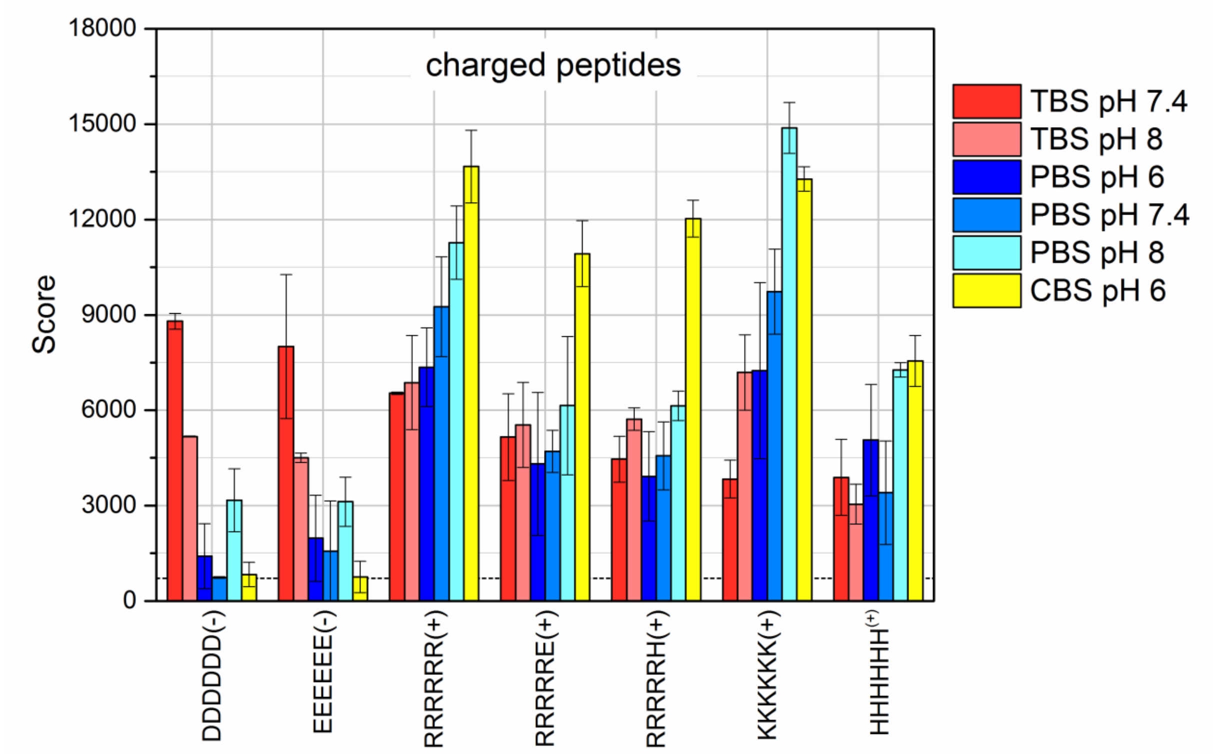

b)

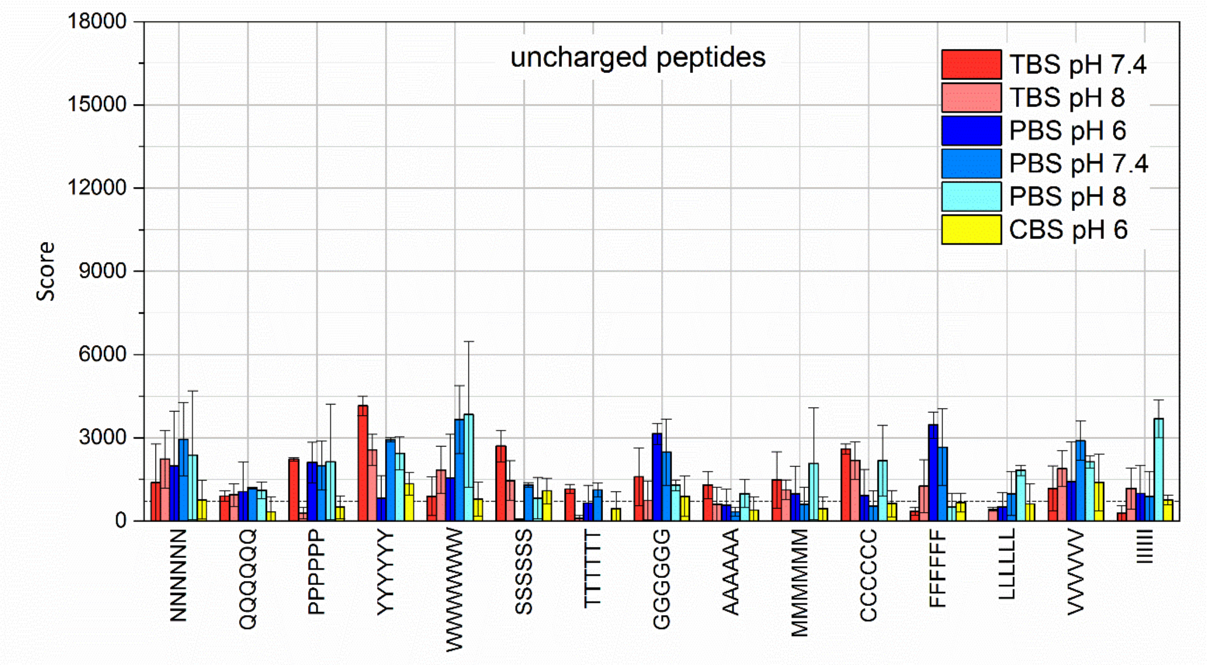

Figure S8 Summary of peptide interaction scores with MNP in different buffer conditions for a) charged peptides and b) uncharged peptides

Derivation of the Electrostatic Interaction Model

To model the interaction of charged peptides to the MNPs, we first determine the ion concentrations present in the solution of the mixture peptides/buffer/surface. The concentration of peptides is several orders of magnitude lower than the concentrations of the nanoparticles and the buffer concentration which is by a factor of 10 higher. Therefore, the concentrations of the different buffer species are independent from the concentrations of peptides and magnetic nanoparticles and can be obtained from the pK_a values for a given pH of the solution.

Reactions of charged amino acids with buffer coated MNPs of opposite charge

Both, charged peptides and buffer ions, can bind the MNPs. We therefore model the concentration of ions and peptides on the MNPs by a set of reaction equilibria. An overall positive charge of the MNPs was observed below pH 7.8, and a negative charge above pH 7.8, however the surfaces can be amphoteric, i.e. have positively, NP^+, and negatively, NP^-,charged spots.

The following reaction steps are assumed for spots oppositely charged to the peptides

1) Charged buffer species bind to the respective positive and negative spots of the nanoparticles

$$NP^- + B^{n+} \rightarrow NPB^{(n-1)+}$$

or

$$NP^+ + B^{n-} \rightarrow NPB^{(n-1)-}$$

where NP^+ denotes a spot on the nanoparticle that carries a unit charge. Reactions with uncharged spots are not considered. If such a “spot” binds to a singly charged ion of opposite charge, the “spot” is effectively neutralized. If it binds to an ion of higher valence, the charge of the spot is effectively inverted. For subsequent binding events with the peptides it is irrelevant whether the charge on the surface is a “spot” originating from the bare nanoparticle or whether it arises from a buffer ion binding to the surface. We therefore quantize the spots on the surface as spots of unit charge of either sign, which can react with the surface (S^+ or S^-).

2) In the second step the nanoparticles coated with buffer and a set of k spots on the nanoparticle each with unit charge are reacting with a charged homopeptide of length l:

$$k\,S^+ + P^{l-} \rightarrow kSP^{(k--l)}$$

where k <= l. An analogous equilibrium exists for peptides/spots of opposite charge.

Considering the reactions given above, the binding strength, and therefore ΔG, depends on the Coulomb interactions of the charges and the above reaction can be generalized to binding all or a fraction of the amino acids comprising the peptide to the surface. Because we consider only the equilibrium of the peptide as a whole with the surface it is sufficient to assume that there is an average set of oppositely charged spots available, which may be smaller than the length of the peptide.

Table S2 Example for the calculation of the binding affinity between the arginine homopeptide and MNPs in the presence of phosphate buffer at pH 6.

	pH = 6			
	Charge	Relative fraction	Charge	Relative fraction
Phosphate buffer (-)	1-	94%	2-	6%
Arg(+)	1+	100%		
Scaling factor: abs(charge(buffer*AA))	1	94%	2+	6%
Calculated binding affinity	(1 * 94) + (2 * 6) = 106			
Binding affinity scaled to the experimental values	6461			

In this way, binding affinities can be calculated for all buffer conditions. The calculated values are assumed to be proportional to the Gibbs free energy and can be compared with the experimentally obtained scores.

Reactions of charged peptides with buffer coated MNPs of the same charge

In case that the peptide and the buffer species have the same charge, adsorption of the peptide competes with adsorption of the buffer species. Here, the equilibrium constants of the two reactions have to be compared. In the following we will present the equations for the example of a singly positively charged buffer ion, which neutralizes the spot it binds to. Reactions for buffer ions of higher valence or negative charge proceed analogously. We note again that the concentration of ions is orders of magnitude higher than the concentration of amino acids. For this reason, the equations can be considered hierarchically.

1) Reaction of buffer and nanoparticles

$$S^- + B^+ \leftrightarrow SB$$

with the equilibrium constant equal to,

$$K_{SB} = \frac{[SB]}{[B^+][S^-{}_0]}$$

Where $[S^-{}_0]$ is the concentration of negative spots on the bare nanoparticle.

2) Second, we consider the reaction of charged homopeptides of length l and the remaining charged spots on the nanoparticles

$$P^{l+} + k\, S^- \leftrightarrow kSP^{l-k}$$

The equilibrium constant is equal to,

$$K = \frac{[kSP^{l-k}]}{[P^{l+}][S^-]^k}$$

In presence of the buffer, the effective concentration of adsorption sites on the MNPs available for reactions the peptide is equal to:

$$[S^-] = [S_0^-] - [SB] = [S_0^-]\left(1 - \frac{[SB]}{[S_0^-]}\right)$$

and

$$\frac{[SB]}{[S_0^-]} = K_{SN}[B^+]$$

Thus,

$$[S^-] = [S_0^-]\left(1 - K_{SB}[B^+]\right)$$

The equilibrium constant is related to the change of the standard Gibbs free energy of the reaction between nanoparticles and amino acids by

$$K = e^{-\frac{\Delta G_{kS^- P^{l+}}}{RT}}$$

The concentration of the nanoparticles bound to the peptides can be expressed by:

$$[\mathrm{k}S^- \mathrm{P}^{l+}] = \mathrm{K}\ [\mathrm{P}^{\mathrm{l}+}][S^-]^k = \mathrm{e}^{-\frac{\Delta G_{kS^- P^{l+}}}{\mathrm{RT}}}[\mathrm{P}^{\mathrm{l}+}]\left([S_0^-](1 - K_{SB}\ [B^+])\right)^k$$

$[\mathrm{P}^{\mathrm{l}+}]$ and $[\mathrm{S}_0^-]$ are unknown, but constant in all experiments. The term on the left is proportional to the loading of the peptide spot, as it corresponds to the amount of bound peptide. The concentration of buffer ions can be determined as indicated above for all experimental conditions. Therefore $\log[\mathrm{k}S^- \mathrm{P}^{l+}] \sim \Delta G_{exp}$ should be proportional to the right hand side of this equation for all experiments (i.e. buffer conditions and pH) if the interactions are purely governed by charge as assumed above. The results of this comparison are indicated in Fig 7 of the manuscript, supporting the assumptions of the model. If the interactions between the different peptides and the surface were strongly influenced by other interactions, there would be no correlation between the observed and the predicted values of the above equation. We can therefore conclude that the experimental data is consistent with the assumption that the interactions are only significantly influenced by the charge of the peptide. We note that we have nowhere assumed that the interactions of the peptide with the surface are additive contributions of the individual amino acids. The effect of the translational and rotational motions on the standard binding free energy should be negligible as described in literature.[2]

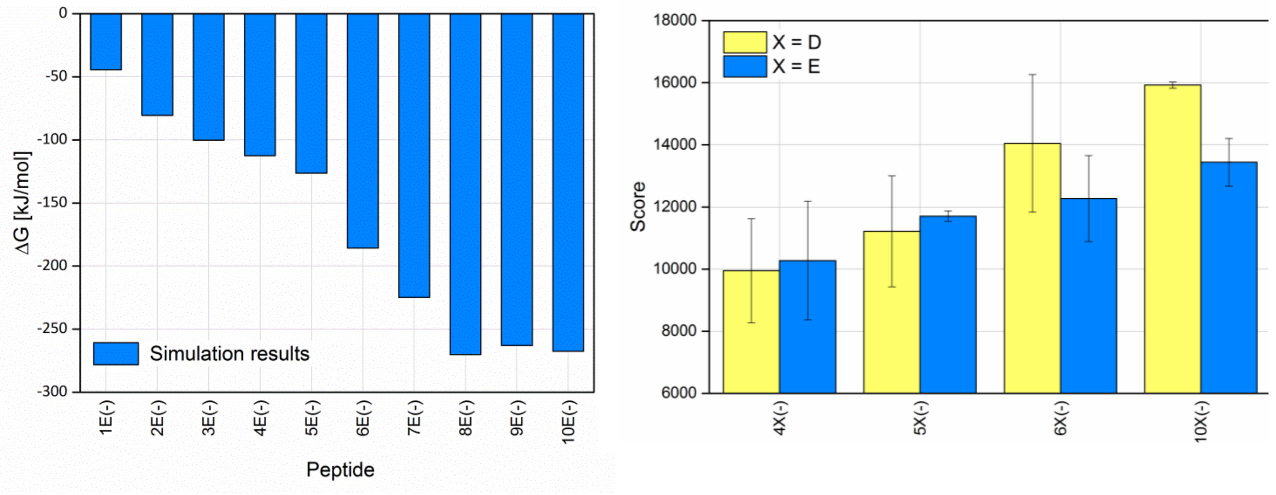

Figure S9 Dependence of homo-peptide interaction with MNP surface from the peptide length determined by Monte Carlo simulation (left) and peptide array experiments (right). The simulation was performed in triplicates and the experimental data stem from two experiments with three replicates each.

To obtain the free energies of binding (ΔG kJ/mol) of homo-peptides with MNP surfaces we performed Monte Carlo simulations with implicit solvent / implicit surface force-field, the Effective Implicit Surface Model (EISM)[3] implemented in the SIMONA package[4]. EISM based simulations are fast and efficient method to evaluate the binding affinity of peptides with inorganic surfaces. The force-field comprises following terms:

$$E = E_{INT} + E_{SLIM} + E_{SLJ} + E_{SASA} + E_{PIT}$$

The term E_{INT} (internal energy) refers to the internal interactions of a peptide i.e., Lennard-Jones (LJ), Coulomb and dihedral terms parametrized by any of the standard force-fields available. In this investigation we have used the AMBER99IDLN* force-field[5]. The second term E_{SLIM} refers to an implicit membrane model SLIM[6] based on a layered Generalized Born model and it is used to model the electrostatic interaction of the peptide with the presence of the surface. SLIM describes the system in terms of different dielectric regions: the peptide itself is assigned a dielectric constant ϵ_c= 1, the surface is modeled as a single dielectric slab with dielectric constant ϵ_h = 34.5 and the solvent region is assigned dielectric constant ϵ_w =80. E_{SLJ} describes the Lennard Jones interactions, between the peptide and the surface. The term E_{SASA} in Eq. 1 (solvent accessible surface area) is used to model the interactions of individual amino acids with the surface that are not accounted for by the previously described interactions. The interaction of the peptide and the surface is modelled to be proportional to the solvent accessible surface (SASA) of the peptide with a residue-specific surface tension, denoted as γ_i.(where i=20 amino acids)

The residue-specific surface tension parameters γ_i (where i = 20 amino acids) for all 20 amino acids for the magnetic surface is computed based on peptide array experiment carried out in Tris buffer at pH =7.4 (Figure 3). We then perform simulations for homopeptides of Argn (Ace-Rn-NMe) and Glun (Ace-En-NMe) homopeptides of varying length (where n = 1-10). The homopeptides are capped at N- and C-terminal with Acetyl and N-methyl group respectively. We perform 4 million simulation steps per simulation at 300 K using metadynamics protocol with the PLUMED plugin[7]. For the metadynamics simulations we use a single dimensional reaction coordinate adding Gaussians of width 0.1 and height 0.005 kJ/mol every 20 simulation step. Reaction co-ordinate in our simulations is distance of the peptide in Z-direct from the surface. We use sum_hills tool in PLUMED to calculate the Gaussians deposited to plot the free-energy. The binding affinity of a particular peptide sequence is characterized by the difference in Gibbs free energy between the bound and the unbound state calculated using EISM-Monte Carlo simulations. To reduce the numerical error, we average the free energy of binding over 20 independent simulations.

References

1. Nelson, D.L. & Cox, M.M. *Lehninger Principles of Biochemistry.* 5th ed. (W. H. Freeman & Company, New York, 2008).

2. Yu, Y. B., Privalov, P. L. & Hodges, R. S. Contribution of Translational and Rotational Motions to Molecular Association in Aqueous Solution. *Biophysical Journal* **81,** 1632–1642 (2001).

3. Anand, P., Borkowska-Panek, M., Gußmann, F., Fink, K. & Wenzel, W., pp. 339–340.

4. Strunk, T. *et al.* SIMONA 1.0: An efficient and versatile framework for stochastic simulations of molecular and nanoscale systems. *J. Comput. Chem.* **33,** 2602–2613 (2012).

5. Lindorff-Larsen, K. *et al.* Improved side-chain torsion potentials for the Amber ff99SB protein force field. *Proteins* **78,** 1950–1958 (2010).

6. Setzler, J., Seith, C., Brieg, M. & Wenzel, W. SLIM: An improved generalized Born implicit membrane model. *J. Comput. Chem.* **35,** 2027–2039 (2014).

7. Tribello, G. A., Bonomi, M., Branduardi, D., Camilloni, C. & Bussi, G. PLUMED 2: New feathers for an old bird. *Comput. Phys. Comm.* **185,** 604–613 (2014).
